# Three-dimensional drip infusion CT cholangiography in patients with suspected obstructive biliary disease: a retrospective analysis of feasibility and adverse reaction to contrast material. 

**DOI:** 10.1186/1471-2342-6-1

**Published:** 2006-04-22

**Authors:** A Persson, N Dahlström, Ö Smedby, TB Brismar

**Affiliations:** 1 Center for Medical Image Science and Visualization (CMIV), Linköping University Hospital, Sweden; 2 Department of Radiology, Hudiksvall Hospital, Sweden; 3 Division of Radiology, Karolinska Institutet, Sweden

## Abstract

**Background:**

Computed Tomography Cholangiography (CTC) is a fast and widely available alternative technique to visualise hepatobiliary disease in patients with an inconclusive ultrasound when MRI cannot be performed. The method has previously been relatively unknown and sparsely used, due to concerns about adverse reactions and about image quality in patients with impaired hepatic function and thus reduced contrast excretion. In this retrospective study, the feasibility and the frequency of adverse reactions of CTC when using a drip infusion scheme based on bilirubin levels were evaluated.

**Methods:**

The medical records of patients who had undergone upper abdominal spiral CT with subsequent three-dimensional rendering of the biliary tract by means of CTC during seven years were retrospectively reviewed regarding serum bilirubin concentration, adverse reaction and presence of visible contrast media in the bile ducts at CT examination. In total, 153 consecutive examinations in 142 patients were reviewed.

**Results:**

Contrast media was observed in the bile ducts at 144 examinations. In 110 examinations, the infusion time had been recorded in the medical records. Among these, 42 examinations had an elevated bilirubin value (>19 umol/L). There were nine patients without contrast excretion; 3 of which had a normal bilirubin value and 6 had an elevated value (25–133 umol/L). Two of the 153 examinations were inconclusive. One subject (0.7%) experienced a minor adverse reaction – a pricking sensation in the face. No other adverse effects were noted.

**Conclusion:**

We conclude that drip infusion CTC with an infusion rate of the biliary contrast agent iotroxate governed by the serum bilirubin value is a feasible and safe alternative to MRC in patients with and without impaired biliary excretion.

In this retrospective study the feasibility and the frequency of adverse reactions when using a drip infusion scheme based on bilirubin levels has been evaluated.

## Background

For diagnosis of hepatobiliary disease, ultrasound and MR cholangiography (MRC) are most frequently used. Endoscopic Retrograde Cholangiography (ERC) is often regarded as the gold standard for visualising biliary disease. The latter modality is invasive, user-dependent and may induce pancreatitis. It should therefore not be performed in patients where intervention is less certain. Ultrasound, on the other hand, is easily tolerated by the patients and cost effective. The modality is, however, user-dependent and the captured images are not easily understood by clinicians. MRC is superior in visualising the biliary system, and the images are appreciated by the surgeons at surgical planning. It does not require any contrast agent to visualise the bile ducts, and dilatation and gallstones in the common bile duct are easily detected [1-3]. Unfortunately, MRC cannot be performed in all patients and hospitals due to limited availability of MRI or due to contraindications. MRC is also often inconclusive in patients with air in the biliary system, e.g. after papillotomy or liver surgery with entero-hepatic anastomoses (such as Whipple's operation and Billroth 2). Surgical clips after cholecystectomy may also give artefacts mimicking a ductal cancer or a stone [4,5]. An alternative non-invasive method to ultrasound and MRC is therefore required.

Computed Tomography Cholangiography (CTC) is a fast and widely available technique to visualise hepatobiliary disease. Without contrast administration, multi detector CT has been reported to have a sensitivity of 65%–88% and a specificity of 84%–97% to detect gallstones [6,7]. Techniques to improve the sensitivity and specificity by administering biliary contrast media orally [8] or intravenously [9,10] have been developed, but are not wide-spread. Possible explanations for infrequent use of CTC might be the low resolution of single detector helical CT and reports of an unacceptable high number of adverse events after injection of meglumine iotroxate [11]. With the development of multidetector CT, the resolution of CTC exceeds that of MR. The number of adverse reactions with biliary contrast media has probably diminished by infusing the contrast media instead of injecting.

The aim of this retrospective study was to evaluate prolonged drip infusion CT cholangiography (CTC) in patients with suspected obstructive biliary disease with respect to both feasibility and rate of adverse reactions after administration of the biliary contrast agent (iotroxate).

## Methods

This is a retrospective study in 142 consecutive patients (68 men and 74 women, mean age 69 years, range 24 – 95 years) referred for investigation of biliary disease during the period from January 1996 to January 2003. After approval by the ethics committee for the region, the medical records of all patients were retrospectively reviewed regarding bilirubin level, infusion time and adverse events. Adverse events were defined as any signs of reaction to contrast media that occurred after the injection, such as anaphylaxis, urticaria and respiratory distress.

### Administration of contrast media

The serum bilirubin concentration was measured before CT examination using standard clinical laboratory methods used at the hospital. 100 ml of meglumine iotroxate (Biliscopin^®^, Schering AG, Berlin, Germany) 50 mg I/ml was administered by intravenous drip infusion. In order to allow longer infusion times, the solution volume was increased by dilution with isotonic sodium chloride (500 ml). The infusion time was determined by the measured bilirubin level according to a schematic protocol (Table 1). Following the guidelines from the manufacturer, the drip infusion was started at a low infusion rate (0.5 ml/min) and increased to the desired infusion rate during the following 3–5 minutes. The CT scan was started immediately after the infusion was completed. For distension of the distal duodenum, the patients ingested two glasses of drinking water immediately before the CT examination. To evaluate compliance to the protocol, the medical records were reviewed regarding the given infusion time at the ward.

### Scanning parameters

Patients were scanned in the right oblique position by means of thin-section single-breath-hold helical CT in the cranio-caudal direction. Specific scan protocols varied depending on the CT scanner available at the time of examination (Table 2). Between December 1995 and November 1999, 102 patients were scanned with a single-slice CT scanner (Somatom A; Siemens Medical Systems, Forcheim, Germany). From December 1999 to November 2002, a 4-slice multi-detector CT scanner (Somatom Volume Zoom; Siemens Medical Systems, Forcheim, Germany) was used in 44 exams. Between December 2002 and January 2003, a 16-slice multi-detector CT scanner (Somatom Sensation16; Siemens Medical Systems, Forcheim, Germany) was used in 6 exams.

### Evaluation of contrast media excretion

The attenuation in choledochus and liver was obtained retrospectively by measurement in the restored digital images in all examinations with bilirubin >19 μmol/L (n = 42), as well as in 67 individuals also described in another study [12], 19 of the latter with bilirubin >19 μmol/L. In total, attenuation values from 90 (= 42 + 67 - 19) patients were obtained.

### Review of literature

A MEDLINE search was performed for all clinical studies in English published during the period 1975–2004 concerning iotroxate, using the words "Biliscopin" or "iotroxate". All articles were reviewed for reports regarding adverse events. The pooled frequency of adverse events was calculated for all articles with a number of patients >100 where the contrast had been infused for 30 minutes or more.

### Statistical methods

Data are given as mean (± standard deviation). Frequencies are given with their 95% confidence interval, computed with normal approximation.

## Results

Out of 153 examinations performed in 142 patients, one subject experienced a minor reaction (pricking sensation in the face) following the administration of 70 ml of contrast. In this patient, the pre-exam bilirubin value was normal (11 μmol/L) and the planned infusion time was 60 minutes. Four weeks later, the same patient successfully underwent a repeated CT cholangiography using the same infusion rate without any adverse reactions. In the other 141 patients (151 examinations), no adverse reaction was noted in the medical records. Thus, the observed frequency of adverse reactions in this material was 1/153 (0.65%). Of the 153 examinations, 10 were performed in out-patients. These patients normally stay in the radiology department one hour after the contrast injection has been completed. Due to the retrospective nature of this study, late mild adverse advents may not have been recorded in these 10 patients. More severe adverse reactions such as a skin rash, itches, etc. are, however, usually reported to the hospital by the patients and according to the routines of the hospital, adverse reactions are always noted in the medical records after an X-ray examination. The hospital is the only one in the district and no notes could be found about adverse advents in the patients' files (files from departments of radiology, surgery and internal medicine were reviewed).

**Figure 1:**
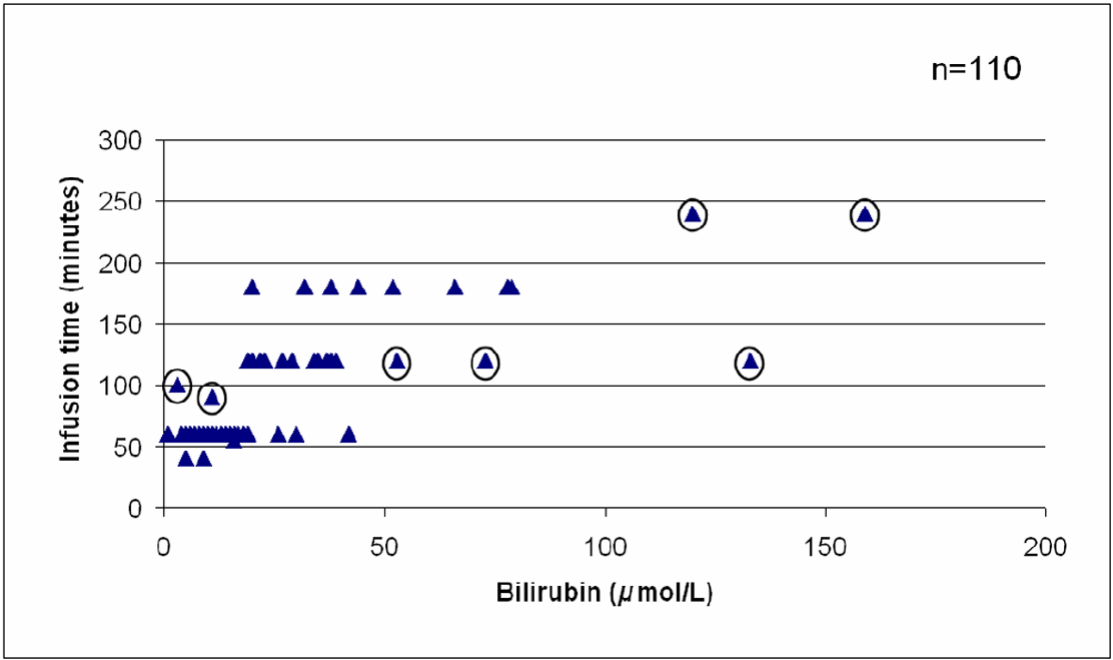
The infusion time of iotroxate (Biliscopin^®^) in relation to bilirubin level prior to the investigation. The recommended infusion times were followed in 103 out of the 110 cases (94%) where information on infusion time was found in the medical records. Cases in which the recommendations were not followed are encircled (n = 7). Unfortunately, none of the three (3/110) examinations with a bilirubin value >100 μmol/ml was performed according to the infusion scheme. The patient with the highest bilirubin value (159 μmol/ml) had good diagnostic excretion of contrast in the bile ducts, whereas the other two had no excretion.

The mean bilirubin value was 20 (± 25) μmol/L. 42 patients had an elevated bilirubin value (defined as >19 μmol/L). Information regarding the infusion time used at the ward had been noted in the medical records in 110 out of 153 examinations. The mean infusion time was 82 (± 42) minutes. Disregarding potential measurement errors of at most 2 μmol/L, seven infusions (5%) had not been performed according to the protocol. Five of these received the infusion too fast and 2 too slow (Fig. 1). All three patients with a bilirubin >100 μmol/L were among the seven patients who did not receive the correct infusion rate. The intended infusion rate (5 hours) could therefore not be evaluated.

Excretion of contrast media was observed in 93% (143/153) of all exams (one examination aborted due to potential contrast reaction). In patients with elevated serum bilirubin (>19 μmol/L) contrast media in the bile was observed in 36 out of 42 patients (86%). No visible secretion of contrast was reported in 9 patients (Table 3). In three of these, the infusion protocol had not been followed, with too fast infusion (bilirubin 73–133 μmol/L). The final diagnoses in the patients with no visible secretion are also shown in Table 3. Three of these had occlusive intraductal stones, all of which were reported at the CTC. Two patients had a malignancy affecting the bile ducts. One of these was reported at CTC and the other showed signs of dilated bile ducts.

The remaining 4 patients had hepatitis, pancreatitis, cholangitis or cholecystitis.

**Figure 2:**
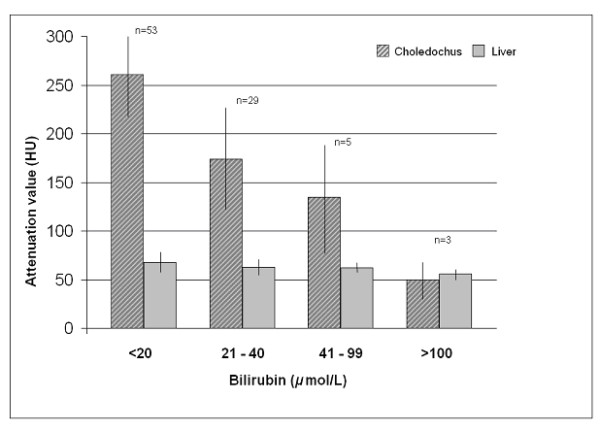
Attenuation in choledochus and liver at DIC-CT as a function of serum bilirubin before the examination.

The observed attenuation in choledochus and liver for different serum bilirubin levels is shown in Fig. 2.

### Review of literature

In total, 42 original publications in English were found. Those with more than 100 patients and with an infusion time of 30 minutes or more are listed in Table 4 as well as the pooled number of adverse events (2.27%).

## Discussion

**Figure 3:**
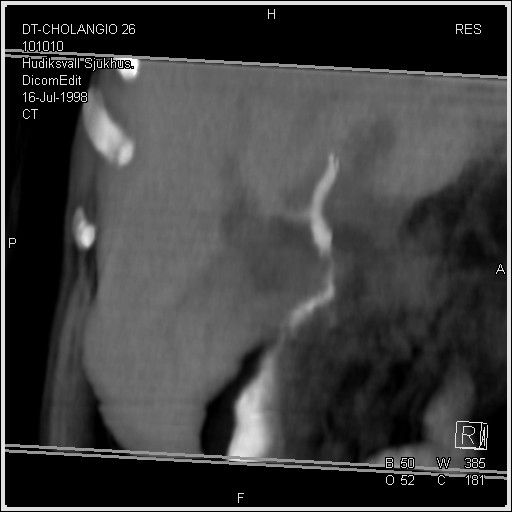
In spite of an elevated bilirubin value, a good contrast excretion can be observed when a prolonged infusion time is used. In this case, the bilirubin value was 78 μmol/L and the infusion time was 3 hours. Final diagnosis was status post choledochoduodenostomy.

**Figure 4:**
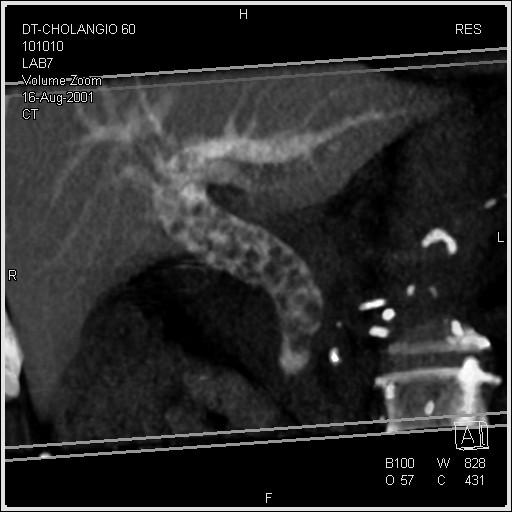
A vast number of biliary stones visualized in the choledochus duct. Pre-examination bilirubin was 29 μmol/L (infusion time not noted in the medical record). ERCP verified the bile duct stones.

When the bile ducts are obstructed, excretion of bile and contrast media is decreased. It has therefore been assumed that CT cholangiography cannot be performed in patients with elevated serum bilirubin concentration [9,10]. In this study, the infusion rate of the contrast media was adjusted to the bilirubin value (Table 1). The aim was to keep the concentration within the excretory capacity of the hepatocytes in order to optimise the concentration in bile. By using this scheme, contrast excretion into the bile ducts was observed in 93% of all exams. In patients with elevated serum bilirubin (>19 μmol/L), contrast media in the bile was observed in 86%. Excretion of contrast media was noted even when the bilirubin concentration was as high as 159 μmol/L (Fig. 3, 4). It has previously been recommended not to perform CTC in patients with bilirubin >50 μmol/L (3 mg/dL) [14]. In this study, excretion was observed in four out of eight patients with bilirubin >50 μmol/L. In three of those without contrast excretion, however, the infusion protocol had not been followed (too fast infusion). Although absence of contrast media in the bile ducts is more likely in patients with greatly elevated bilirubin, CTC is not useless in these patients – only two of the nine examinations without contrast excretion were inconclusive. In the other seven cases, CTC findings could guide the referring physician to other examinations and the final diagnosis (Table 3).

The lack of excretion may also constitute valuable information. Patients without excretion are likely to have either a total occlusion of the main bile duct/choledochus or severely impaired hepatocyte function. The bilirubin concentration, if not already considerably elevated, is likely to increase in these patients. In this study, the lack of excretion could be explained by the final diagnosis in all patients (Table 3).

The protein-binding characteristics essential for biliary contrast media increase the risk of adverse reactions [11,15]. In a previously published review of the literature on the frequency of adverse reactions in examinations with short injection time (<10 min), the pooled number of adverse events was three times higher (16% vs. 5%) than after infusion (>30 min) of the same amount of contrast media [11]. The frequency of adverse events of iotroxate (Biliscopin^®^) at infusion has been reported to be as high as 3.4%, with a pooled frequency of 1.9% (Table 4). It has been proposed that the tolerance of intravenous biliary contrast media is improved when a slow infusion technique is used (up to one hour of infusion) [16,17]. Our study supports this proposal, as there was only one adverse reaction, which was mild, in 142 patients and 153 examinations (0.65%).

After an inconclusive ultrasound examination, MRC has the advantage of not exposing the patient to radiation and contrast media. On the other hand, in many clinical situations the availability of MRI examinations with short notice is limited. The sensitivity and specificity of contrast enhanced CTC to detect choledocholithiasis are comparable to those of MRC (sensitivity 86–93% and 80–95%, respectively, and specificity 94–100% and 88–96%, respectively) [3,8,18,19]. CTC is also faster than MRC, which may be of importance in patients with difficulties in lying in the supine position for a prolonged time or when evaluating severely ill patients.

CTC has been used to preoperatively evaluate aberrant bile ducts before laparoscopic cholecystectomy. The frequency of anatomic variants that may affect the outcome of laparoscopic cholecystectomy was estimated to be 15% with CTC [16,20,21]. In clinical practise, the frequency of bile duct injuries is about 0.5–1.5% [22,23]. The potential value of pre-operative mapping of the biliary system by using CTC must therefore be weighed against the cost and radiation.

With injection of contrast media via biliary drainage catheters, CTC has been successfully used to visualize the extent of ductal invasion by hilar carcinoma [24]. Whether administration of contrast media orally or intravenously may achieve similar results in patients without drainage has not been shown.

**Figure 5:**
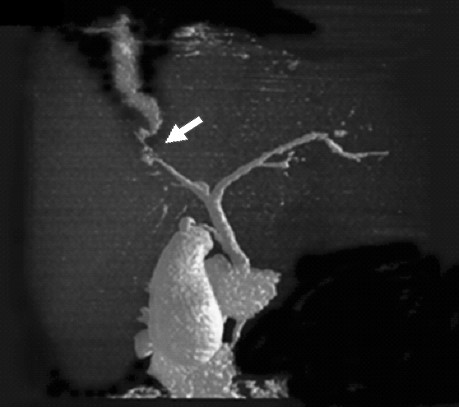
Traffic accident with a liver rupture and leakage from a small bile duct. The DIC-CT examination led the surgeon correctly to the leaking bile duct (arrow). The diameter of the ruptured bile duct was 1 mm.

In clinical practise, CTC has been reported to be the preferred modality to evaluate living donors prior to liver transplantation [25]. The main reported advantage to MRI and MRC was the superior mapping of the biliary tree [26] – in other respects the two modalities were considered equivalent for the planning [25]. After introduction of CTC, the use of an intraoperative cholangiogram has been reported to be significantly reduced at living donor liver transplantation [27]. After liver surgery, there is a risk of biliary leaks. In these patients, and in patients with traumatic rupture of the biliary tree, CTC may be useful to demonstrate the leak (fig 5) [28]. Cholescintigraphy can also demonstrate biliary leaks [29], but the resolution is low compared to CT. To our knowledge, there is no other non-invasive technique to visualise a leakage from the biliary tree (unless a functioning external biliary drainage is present, enabling a secondary cholangiography).

CTC has been shown to provide kinetic and functional information [30]. This is also possible in contrast enhanced MRC when hepatocyte specific contrast media is used (Gd-BOPTA and Gd-EOB-BOPTA). Further studies are required to evaluate whether contrast enhanced MRC or CTC should be preferred to evaluate biliary kinetics.

The main disadvantages of CTC compared to MRC are the use of radiation and contrast media. In our study, including the literature review, the frequency and severity of adverse reactions was low when infusing the contrast media. The disadvantage of infusion is the need of supervision, which is impractical in a radiology department. In our setting, this problem was solved by admitting the patients to a ward for infusion prior to CTC.

## Conclusion

This study indicates that drip infusion CTC with an infusion rate of iotroxate governed by the serum bilirubin concentration is a feasible and safe tool in patients with and without impaired biliary excretion. In addition to those with inconclusive MRC or contraindications, CTC is a diagnostic alternative in patients already admitted to the hospital for whom a reliable diagnosis or mapping of the biliary tree is required within a limited time. In younger patients, non-ionising methods (i.e. MRC or a repeated ultrasound examination) should be preferred.

## Competing interests

The author(s) are not aware of any conflicts of interest relating to this article. For the linguistic revision of this manuscript AP received from Schering AG a support equalling to 300 Euro.

## Authors' contributions

AP was responsible for study design, data collection, was one of the viewers, performed literature search and manuscript preparation. ND was one of the viewers. ÖS was responsible for study design and statistical analyses. TB was responsible for study design, was one of the viewers, performed literature search and finalized manuscript. All authors contributed during manuscript preparation, and read and approved the final manuscript.

## Pre-publication history

The pre-publication history for this paper can be accessed here:



## Figures and Tables

**Table 1:** The infusion rate of iotroxate (Biliscopin^®^) was governed by the bilirubin level prior to the investigation. The same total amount of Iodine (5 g) was given to all patients.

Serum bilirubin	Infusion time
<20 μmol/ml	40–60 min
21–40 μmol/ml	1–3 hours
41–99 μmol/ml	3–4 hours
>100 μmol/ml	5 hours

**Table 2:** CT acquisition parameters

**Type of scanner**	**Collimation**	**Pitch**	**Increment**	**mAs**	**kV**	**Number of examinations**
Single Slice	1 × 5.0 mm	1.5	1 mm	200	120	103
Multi-slice	4 × 2.5 mm	6	1 mm	130	120	46
Multi-slice	16 × 0.75 mm	varying	0.5 mm	130	120	4

**Table 3:** The bilirubin value, infusion time and final diagnosis in the nine cases where no secretion of contrast media was observed at DIC-CT.

Bilirubin value, μmol/L	Infusion time, minutes	Reported findings in medical records, final diagnosis	Reported findings at DIC-CT
**133**	120	Hepatitis Type B^1^	wide bile duct
**120**	240	Pancreatitis ^1,2^	inconclusive
**79**	180	Intraductal stone in choledochus and pancreatitis ^7,2,6^	Intraductal stone
**73**	120	Cholecystitis^1,2^	wide bile duct
**30**	unknown	Concrememt in choledochus^1,2,3,6,^	Intraductal stone
**25**	unknown	Concrement in choledochus, Total occlusion and Klatskin tumour^5,7^	Intraductal stone, tumour
**16**	60	Distal stenosis in choledochus and pancreatitis ^4,5,7^	inconclusive
**12**	60	Total occlusion in choledochus, pancreas tumour ^2,3^	wide bile duct
**8**	60	Post operative cholangitis/cholecystitis with bile fistula and leakage^1,6,7^	fluid-filled cavity

The method by which the final diagnosis was made is indicated by the superscript numbers where 1 = laboratory findings, 2 = ultrasound, 3 = ultrasound with fine needle biopsy, 4 = MRCP, 5 = operation, 6 = ERCP and 7 = PTC.

**Table 4:** Published studies on the frequency of adverse reactions at infusion of iotroxate at intravenous cholangiography. Included are all studies with at least 100 patients using an infusion time of at least 30 min. The severity of the reactions is graded as reported. The number in superscript denotes the corresponding reference.

	**No. of Patients**	**Total**	**Minor**	**Intermediate**	**Severe**	**Fatal**
Nilsson 1987^11^	1 446	49 (3.4%)	41 (2.9%)	5 (0.35%)	3 (0.21%)	0
Daly 1987^31^	286	4 (1.4%)	4 (1.4%)	0	0	0
Joyce 1991^32^	100	2 (2.0%)	2 (2.0%)	0	0	0
Wigmore 1993^33^	100	0	0	0	0	0
Patel 1993^34^	113	3 (2.7%)	3 (2.7%)	0	0	0
Grunshaw 1993^35^	137	4 (2.9%)	3 (2.2%)	1 (0.7%)	0	0
^A^Sacharias 1995^36^	1 061	11 (1.0%)		11 ^A ^(1.0%)	0	0
Kwon 1998^16^	440	2 (0.5%)	2 (0.5%)	0	0	0
Kitami 2006^37^	220	3 (1.4%)	3 (1.5%)	0	0	0
Okada 2005^17^	432	4 (0.9%	4 (0.9%)	0	0	0
Hirao 2000^22^	120	2 (1.7%)	2 (1.7%)	0	0	0
^B^Takamatsu 2004^38^	132	1 (0.8%)	1 (0.8%)	0	0	0
**Total**	4587	85	65	17	3	0
Frequency (95% confidence limits)		1.9% (1.5%–2.2%)	1.4% (1.1%–1.8%	0.4% (0.2%–0.5%)	0.1% (0–0.1%)	0

^A^No difference was made between minor and intermediate adverse events.

^B ^The infusion time was 25–30 min. The number of complications in the article was reported by personal communication.
